# Association between serum S100A9 levels and liver necroinflammation in chronic hepatitis B

**DOI:** 10.1186/s12967-018-1462-2

**Published:** 2018-04-03

**Authors:** Rui Wu, Yuhong Zhang, Yu Xiang, Yishu Tang, Fang Cui, Ju Cao, Lan Zhou, Yan You, Liang Duan

**Affiliations:** 1 Department of Laboratory Medicine, The First Affiliated Hospital of Chongqing Medical University, No.1 You Yi Road, Yuan Jia Gang, Yu Zhong District, Chongqing, 400016 China; 2 Department of Laboratory Medicine, The Second Affiliated Hospital of Chongqing Medical University, No.74 linjiang Road, Yu Zhong District, Chongqing, 400010 China; 3 Key Laboratory of Diagnostic Medicine Designated by the Chinese Ministry of Education, Chongqing Medical University, 400016 Chongqing, China; 4 Department of Pathology, The Second Affiliated Hospital of Chongqing Medical University, 400010 Chongqing, China

**Keywords:** Hepatitis B virus, Damage associated molecular pattern, S100A9, Liver necroinflammation

## Abstract

**Background:**

S100A9 protein, which is recently classified as a novel damage associated molecular pattern, is released from stressed cells undergoing necrosis or secreted by living cells undergoing a stress that act as endogenous danger signal associated with infection, tissue damage and cancer. Here, we evaluated the relationship of serum S100A9 with viral replication and liver necroinflammation in patients with chronic hepatitis B (CHB) infection.

**Methods:**

A total of one hundred and eighty-three recruited patients with CHB infection underwent liver biopsy for grading of necroinflammation (G) and staging of fibrosis (S). Forty-nine healthy individuals were included as healthy controls (HCs). Serum S100A9 levels were determined by enzyme-linked immunosorbent assay. Correlations of serum S100A9 with viral replication and liver necroinflammation were analyzed. The receiver operating characteristic curve was used to assess the discriminating power of serum S100A9 to grade liver necroinflammation (G). Liver normal L02 cells were transfected with a HBV plasmid, and S100A9 levels were determined.

**Results:**

Serum S100A9 levels were increased in CHB patients compared to HCs. Intrahepatic immunoreactivity for S100A9 was enhanced in liver sample from CHB patients. Infection of HBV also resulted in an elevated S100A9 expression in L02 cells. Serum S100A9 was correlated with the serum HBV DNA levels. CHB patients with moderate-to-severe liver necroinflammation (G ≥ 2) showed significantly higher serum S100A9 levels than those without or with mild necroinflammation (G < 2). In patients with normal ALT levels, the area under the curve (AUC) of S100A9 for discriminating patients with moderate-to-severe necroinflammation (G ≥ 2) was 0.791 [95% confidence interval (CI), 0.670–0.913] with 91.7% sensitivity, 65.0% specificity and 78.3% accuracy. In patients with an alanine aminotransferase (ALT) < 2 upper limit of normal, the AUC of S100A9 for discriminating patients with moderate-to-severe necroinflammation (G ≥ 2) was 0.826 (95% CI, 0.729–0.923) with 87.9% sensitivity, 72.5% specificity and 80.2% accuracy.

**Conclusions:**

HBV infection may enhance S100A9 expression. Serum S100A9 levels are correlated with viral load. Serum S100A9 has potential to discriminate the grades of liver necroinflammation, particularly in CHB patients with normal or mildly increased ALT levels.

## Background

Hepatitis B virus (HBV) infection is a common and serious public health concern worldwide [1]. China has a high prevalence of HBV infection, and about 400 million people worldwide are HBV carriers [2]. Once the infection becomes chronic, CHB patients are at greater risk of developing liver cirrhosis, subsequent liver decompensation or hepatocellular carcinoma (HCC) [3].

Serum ALT is the most commonly used marker that reflects the extent of liver necroinflammation [4]. The clinical diagnosis of active CHB and the timing of antiviral therapy are also predominantly based on whether the increased ALT in CHB patients is persistent or intermittent [5]. However, it is different to decide whether the patients commence therapy when they have a high viral load but with normal ALT or an ALT < two ULN. Liver biopsy is currently the gold standard for assessment of HBV-induced liver injury severity or monitoring of CHB progression. Since it is an invasive and painful procedure, liver biopsy limits the application in asymptomatic patients [6]. Additionally, the accuracy of liver biopsies may be affected by the observer variability or sampling error, which may lead to the under-staging of the pathology [7, 8]. Therefore, there is a urgent need for additional promising serum biomarkers for assist in the detection of liver necroinflammation.

Recently, the concept of DAMP has emerged as a novel mechanism for the initiating and promoting the inflammation. DAMPs are molecules released by stressed cells undergoing necrosis or secreted by living cells undergoing a life-threatening stress that act as endogenous danger signals associated with infection, cellular stress, tissue damage and cancer [9, 10]. In the liver diseases, endogenous DAMPs are released by stressed and dying hepatocytes, which alarm the immune system through their potential pattern recognition receptors and related signaling pathways, orchestrate the influx of diverse inflammatory cytokines, and ultimately amplify liver destruction [11]. Notably, DAMPs in plasma or serum has been proved to be an emerging field for noninvasive molecular diagnosis. Increasing evidence suggests that increased serum levels of these DAMPs have been associated with many inflammatory diseases, including sepsis, pancreatitis, arthritis, Crohn’s disease and cancer [12–16]. Therapeutic strategies are being developed to modulate the expression of these DAMPs as well as the inflammatory responses triggered by DAMPs for improving the clinical management of infection- and injury-elicited inflammatory diseases.

S100A9 protein (also known as Calgranulin B or MRP-14), which is recently classified as a noval DAMP, is released from undamaged or infected cells to activate the toll-like receptor 4/myeloid differentiation primary response 88 (TLR4/MyD88) or receptor for advanced glycation end product (RAGE) signaling pathway for induction of innate and inflammatory responses [17]. As an intracellular calcium-binding molecule, S100A9 has a role in migration and cytoskeletal metabolism [18]. Remarkably, cell damage or activation of inflammatory cells triggers its release into the extracellular space where it becomes danger signal that exhibits proinflammatory functions, including potent chemotactic biological function for neutrophils and mononuclear cells and activation of neutrophils [19]. Also, S100A9 is one of the most abundant proteins found in neutrophils, macrophages, and epithelial cells during chronic inflammation [20]. However, to our knowledge to date, whether S100A9 can function as a host-derived molecular pattern during HBV infection and progression is not known.

Since the pathogenesis of liver diseases varies and the role of S100A9 in CHB have not been clarified, serum S100A9 levels from patients with CHB were measured in the present study, and their association with liver disease progression was also analyzed in detail.

## Methods

### Patient samples

Serum samples with chronic HBV infection were prospectively recruited from the First and Second Affiliated Hospital of Chongqing Medical University from May 2015 to May 2017. One hundred and eighty-three treatment-naive patients with CHB were included in this study and underwent liver biopsy. Patients were not included if they were detected with other causes of liver disease, such as superinfection with hepatitis virus C, A, D, and E, autoimmune liver disease and alcoholic liver disease. The patients were consisted of four subgroups [HBeAg(−) inactive HBV carrier, HBeAg(−) immune reactivation phase, HBeAg(+) immune-tolerant phase and HBeAg(+) immune-active phase] at different stages of the natural course of chronic HBV infection according to the American Association for the Study of Liver Diseases (AASLD) [21]. Additionally, forty-nine age and gender-matched healthy volunteers were enrolled as healthy controls (HCs). Also, five normal liver samples were obtained form healthy controls that underwent liver biopsy for excluding malignancy. Informed written consent was obtained from all patients and the study was approved by the Institutional Ethics Committee for human studies at the First and Second hospital affiliated to Chongqing Medical University, Chongqing, China. All procedures were in accordance with the declaration of Helsinki. Patient characteristics are summarized in Table 1.

**Table 1:** The characteristics of enrolled individuals

Parameters	CHB (n = 183)				HCs (n = 49)	p value
	HBeAg(−)		HBeAg(+)			CHB vs HCs
	Inactive carrier (n = 13)	Immune reactivation (n = 79)	Immune-tolerant (n = 23)	Immune-active phase (n = 68)		
Gender (male, %)	10 (76.9%)	57 (72.1%)	13 (56.5%)	38 (55.8%)	36 (73.4%)	> 0.05
Age (years)	37 (9)	47 (19)	39 (10)	41 (12)	37 (14)	> 0.05
ALT (U/L)	23 (19.5)	54 (56)	34 (15)	145 (314)	19 (16.8)	< 0.01
AST (U/L)	24 (11.5)	52 (46)	21 (9)	113 (208)	21 (15.4)	< 0.01
HBV DNA (log_10_ IU/ml)	2.58 (1.95)	4.3 (2.45)	6.32 (1.79)	5.48 (2.17)	N/A	
HBsAg (log_10_ IU/ml)	3.02 (0.57)	3.34 (0.97)	4.02 (0.95)	4.63 (1.7)	N/A	
Grading of necroinflammation (n) G0/G1/G2/G3/G4	9/4/0/0/0	10/37/16/12/4	4/19/0/0/0	1/1/22/22/22		
Stage of fibrosis (n) S0/S1/S2/S3/S4 (S4: cirrhosis)	8/5/0/0/0	5/16/25/23/10	7/16/0/0/0/	2/7/24/22/13		

For age, ALT, AST, HBV DNA titres and HBsAg, data are presented as median (interquartile range)

*N/A* not available

p values < 0.05 are considered as significant

### Blood sampling

Blood samples were first centrifuged at 3000×*g* for 10 min at 4 °C. The serum was removed and recentrifuged at 3000×*g* for an additional 10 min at 4 °C to remove any remaining cellular debris. Finally, aliquoted serum samples were stored at − 80 °C until further processing.

### Liver histology

All liver biopsy specimens were fixed in 10% formalin, embedded in paraffin, and stained with either hematoxylin and eosin or masson’s trichrome staining. The sections were then independently examined by two experienced pathologists who were unaware of clinical status. Histologic examination of necroinflammatory lesions and fibrosis was evaluated based on the modified scheuer cooling system [22].

### Serological test and measurement of serum S100A9

All serum samples collected were first blinded and then tested in duplicate. The Alanine aminotransferase (ALT) and aspartate aminotransferase (AST) in serum were assayed using an automated chemical analyzer Hitachi7600-110 (Japan). HBsAg, HBeAg, and antibodies against HBsAg (anti-HBs), HBeAg (anti-HBe) and hepatitis B core antigen (anti-HBc) were determined using the Abbatt i2000 Immunoassay Analyzer. HBV DNA was quantified using by Roche cobas Z480 Analyzer. S100A9 was measured using a human S100A9 ELISA kit (DGE10839, China) according to the manufacturer’s recommended procedure.

### Cell culture and transfection

Human normal liver cell line L02 was purchased from ATCC (American Type Culture Collection, Manassas, VA) and maintained in the Dulbecco’s modified Eagle’s medium (DMEM) with 10% fetal bovine serum (FBS, Hyclone, USA). Cell culture was maintained at 37 °C in a humid atmosphere containing 5% CO_2_. Transfection of L02 cells with pcDNA3.1-HBV or its control pcDNA3.1 was carried out using Lipofectamine 2000 (Invitrogen; USA), and the cells were collected after transfection for indicated time for the subsequent experiment.

### Immunohistochemical (IHC) procedures

The expression of S100A9 in tissues was examined by IHC. The sections from the formalin fixed, paraffin-embedded tissues were deparaffinized and dehydrated. Then the sections were boiled for 10 min in 0.01 M citrate buffer and incubated with 0.3% hydrogen peroxide (H_2_O_2_) in methanol for 15 min to block endogenous peroxidase. The sections were then incubated with the anti-S100A9 polyclonal antibody (1:300 dilution; ab63818, abcam, UK) overnight at 4 °C, following incubation with secondary antibody tagged with the peroxidase enzyme (SP-9000, Zhongshan Golden Bridge, China) for 30 min at room temperature and were visualized with 0.05% 3,3-diamino-benzidine tetrachloride (DAB) till the desired brown reaction product was obtained. The sections were finally counter-stained with hematoxylin. Control sections were performed using phosphate buffer solution (PBS) without a primary antibody. All slides were observed under a Nikon E400 Light microscope and representative images were taken.

### Immunofluorescent staining

Cells were washed with PBS and fixed in 4% paraformaldehyde, then permeabilized with 0.2% Triton X-100. Cover slips were rinsed and incubated with blocking serum and then incubated overnight at 4 °C with primary anti-S100A9 antibody. The cells were then washed with PBS and stained with the corresponding FITC-conjugated secondary antibody. The nuclei were visualized by staining the cells with DAPI. The fluorescent images were then observed and analyzed by fluorescence microscopy.

### Real time quantitative PCR analysis

L02 cells were transfected with HBV for 24 h and then lysed with Trizol (Invitrogen, Carlsbad, CA, USA). Complementary single-stranded DNA was synthesized from total RNA by reverse transcription (TaKaRa, Japan). Primers were also synthesized by Invitrogen. Real time PCR was performed using SYBR Green master mix (TaKaRa, Japan). Quantification of cDNA targets was performed on Roche cobas Z480 Analyzer. GAPDH was used as an internal control. The PCR conditions and primers sequence were as follows: human S100A9 primers (forward) 5′-TCATCAACACCTTCCACCAA-3′ and (reverse) 5′-TTAGCCTCGCCATCAGCA-3′; GAPDH primers (forward) 5′-CAGCGACACCCACTCCTC-3′ and (reverse) 5′-TGAGGTCCACCACCCTGT-3′. Gene expression was determined by normalization against GAPDH expression.

### Western blot assay

Western blot analysis was applied to evaluate levels of S100A9 in cells. Briefly, the cells were collected and washed with ice-cold PBS, then lysed on ice in radio immunoprecipitation assay (RIPA) buffer. Samples containing equal amount of proteins were separated in 10% SDS-PAGE and blotted onto the PVDF membranes. Then the membranes were blocked with 5% bovine serum albumin and incubated with anti-S100A9 or anti-β-actin antibody (1:1000 dilution, respectively), followed by incubation with secondary antibody conjugated with horseradish peroxidase. The proteins of interest were detected using the SuperSignal West Pico Chemiluminescent Substrate kit. The results were recorded by the Bio-Rad Electrophoresis Documentation (Gel Doc 1000, Bio-Rad, USA) and Quantity One Version 4.5.0.

### Statistical analysis

Data were analyzed using SPSS 17.0 (IBM, Armonk, NY). The Kruskal–Wallis or Mann–Whitney test was performed to determine the significance of serum S100A9 levels. Receiver operating characteristic (ROC) curves were generated to classify patients in different groups, as well as for the evaluation of the diagnostic potential of serum S100A9 via calculation of the area under the ROC curve (AUC), sensitivity and specificity according to standard formulas. Correlation coefficients (r) were calculated using spearman correlation. Differences between multiple groups in L02 cells were evaluated using one-way analysis of variance. A p value < 0.05 was considered statistically significant.

## Results

### S100A9 levels in different groups of patients with CHB

**Fig. 1:**
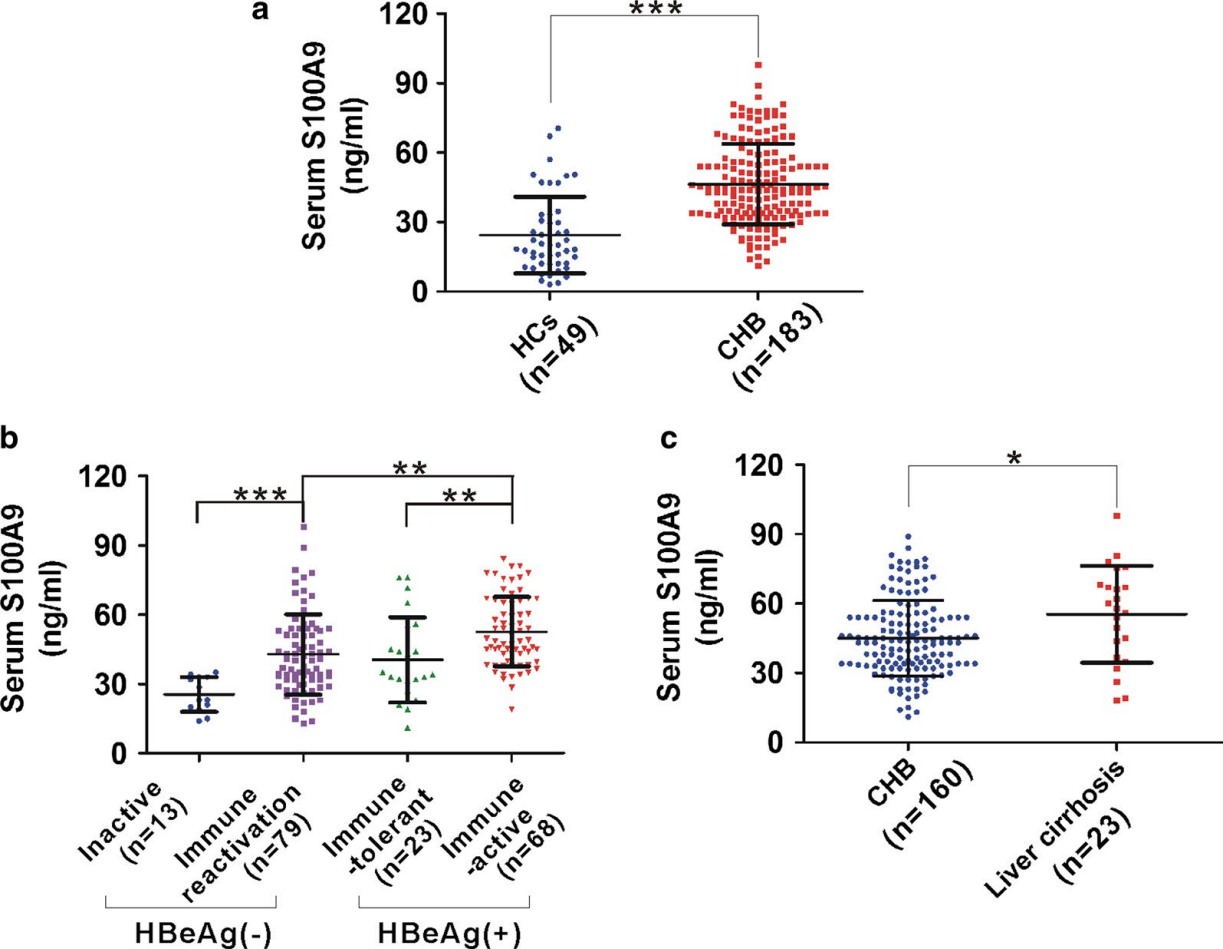
Serum S100A9 levels in healthy subjects and patients samples. **a** ELISA analysis of S100A9 levels from blood serum samples in healthy controls (HCs) and CHB patients. **b** ELISA analysis of S100A9 levels from blood serum samples in four subgroups of CHB patients. **c** ELISA analysis of S100A9 levels from blood serum samples in CHB patients with and without liver cirrhosis. Data represents the mean ± SD, *p < 0.05; **p < 0.01; ***p < 0.001

To assess whether serum S100A9 levels are abnormally altered in CHB patients, we detected and analyzed its levels in different groups of patients and HCs. CHB patients showed significantly higher serum S100A9 levels than HCs (Fig. 1a). According to AASLD guidelines, CHB patients were classified to four subgroups including HBeAg(−) inactive CHB phase, HBeAg(−) immune reactivation phase, HBeAg(+) immune-tolerant phase, HBeAg(+) immune-active phase. We then assessed S100A9 levels in these four subgroups and compared them to each other. In HBeAg(−) two subgroups, S100A9 levels were significantly higher in immune reactivation phase than that in inactive phase (Fig. 1b). In HBeAg(+) two subgroups, S100A9 levels were also higher in immune-active phase than that in immune-tolerant phase (Fig. 1b). Also, S100A9 levels were also higher in HBeAg(+) immune-active phase than that in HBeAg(−) reactivation phase (Fig. 1b). Further, we also detected and analyzed the S100A9 levels in CHB patients with and without liver cirrhosis. CHB patients with liver cirrhosis had higher S100A9 levels than CHB patients without cirrhosis (Fig. 1c).

### Correlation of serum S100A9 with viral replication in CHB patients

**Fig. 2:**
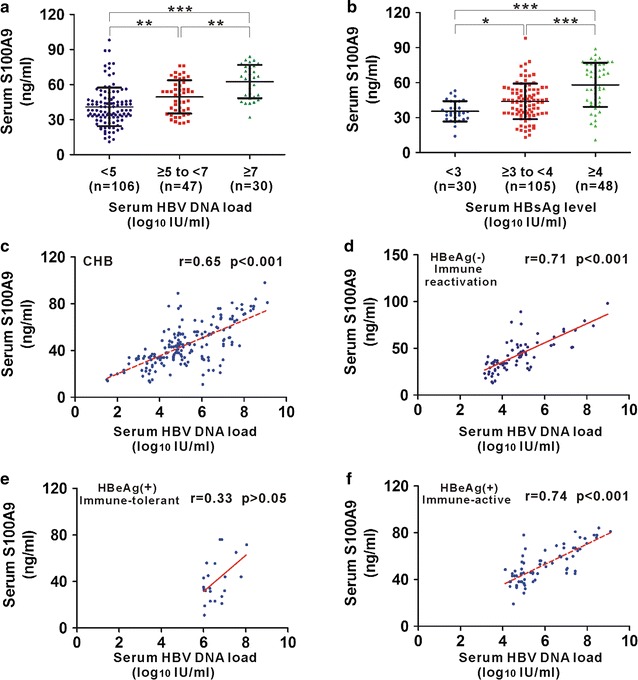
Correlations of serum S100A9 levels with HBV DNA and HBsAg. **a** Distribution of serum S100A9 levels in patients with different viral load (< 5, ≥ 5–7 and ≥ 7 log_10_ IU/ml). **b** Distribution of serum S100A9 levels in patients with different HBsAg levels (< 3, ≥ 3–4 and ≥ 4 log_10_ IU/m). **c** Correlation between serum S100A9 levels and HBV DNA levels in CHB patients. **d** Correlation between serum S100A9 levels and HBV DNA levels in subgroup of HBeAg(−) immune reactivation CHB patients. **e** Correlation between serum S100A9 levels and HBV DNA levels in subgroup of HBeAg(+) immune-tolerant CHB patients. **f** Correlation between serum S100A9 levels and HBV DNA levels in subgroup of HBeAg(+) immune-active CHB patients. Data represents the mean ± SD, *p < 0.05; **p < 0.01; ***p < 0.001

To investigate whether serum S100A9 levels are associated with HBV replication, we analyzed serum S100A9 levels in various viral loads in CHB patients. We found that the increased average level of serum S100A9 was proportional to the increase of serum HBV DNA and HBsAg (Fig. 2a and b). CHB patients with HBV DNA load ≥ 7 log_10_ IU/ml showed higher serum S100A9 levels than the patients with HBV DNA load ≥ 5–7 log_10_ IU/ml or < 5 log_10_ IU/ml. CHB patients with viral load < 5 log_10_ IU/ml showed the lowest average serum S100A9 levels (Fig. 2a). Further, we also analyzed the correlation between serum S100A9 levels and HBsAg levels, a known biomarker of viral replication. We found that S100A9 levels increased significantly with those of HBsAg (Fig. 2b). CHB patients with HBsAg levels ≥ 4 log_10_ IU/ml showed the higher S100A9 levels than the patients with HBsAg levels ≥ 3–4 log_10_ IU/ml or < 3 log_10_ IU/ml. We further analyzed the correlation of serum S100A9 levels with viral loads. S100A9 levels were found to be correlated with HBV DNA levels in CHB patients (Fig. 2c). Since increased levels of HBV DNA are mainly existed in subgroups of HBeAg(−) immune reactivation phase, HBeAg(+) immune-tolerant phase and HBeAg(+) immune-active phase, correlation of serum S100A9 levels and HBV DNA levels was also analyzed in these three subgroups. Positive correlation of serum S100A9 levels with viral loads was found in HBeAg(−) immune reactivation phase and HBeAg(+) immune-active phase but not in HBeAg(+) immune-tolerant phase (Fig. 2d–f).

### Relationship of between serum S100A9 and necroinflammation in CHB patients

**Fig. 3:**
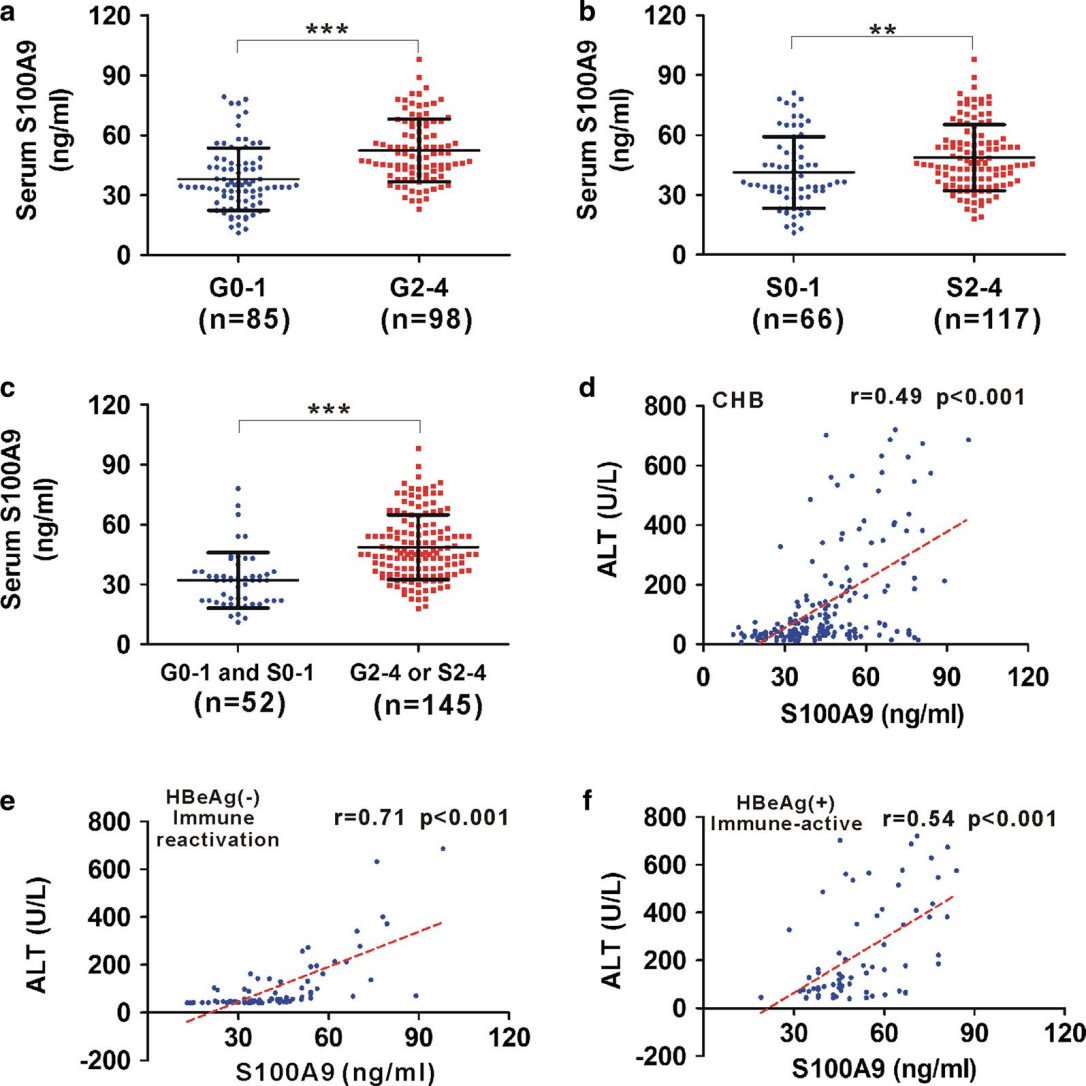
Relationship between serum S100A9 levels and liver necroinflammation or fibrosis. **a** Distribution of serum S100A9 levels in CHB patients with different phases (no or mild liver necroinflammation, G0–1; moderate-to-severe necroinflammation, G2–4). b Distribution of serum S100A9 levels in CHB patients with different phases of fibrosis (no or mild liver fibrosis, S0–1; moderate-to-severe fibrosis, S2–4). **c** Distribution of serum S100A9 levels in CHB patients with different degrees of liver damage (no or mild liver damage, G0–1 or S0–1; significant liver damage, G2–4 or S2–4). **d** Correlation between serum S100A9 levels and ALT levels in CHB patients. **e** Correlation of serum S100A9 levels with ALT levels in subgroup of HBeAg(−) immune reactivation CHB patients. **f** Correlation between serum S100A9 levels and ALT levels in subgroup of HBeAg(+) immune-active CHB patients. Data represents the mean ± SD, *p < 0.05; **p < 0.01; ***p < 0.001

Patients with moderate-to-severe necroinflammation (G2–4) showed higher S100A9 levels than those with no or mild liver necroinflammation (G0–1) (Fig. 3a). Similarly, patients with moderate-to-severe fibrosis (S2–4) also showed higher S100A9 levels than those with no or mild liver fibrosis (S0–1) (Fig. 3b). When combing liver neuroinflammation and fibrosis, patients with significant liver damage (G2–4 or S2–4) showed significantly higher S100A9 levels than those with no and mild liver damage (G0–1 and S0–1) (Fig. 3c). We further analyzed the correlation of serum S100A9 levels with necroinflammation parameter ALT. S100A9 levels were found to be correlated with ALT in CHB patients (Fig. 3d). Since elevated levels of ALT are mainly existed in two subgroups of HBeAg(−) immune reactivation phase and HBeAg(+) immune-active phase, correlation of serum S100A9 levels with ALT in these two phases was also analyzed. S100A9 levels were also found to be correlated with ALT in these two subgroups (Fig. 3e and f).

### Differentiating power of S100A9 for liver necroinflammation

**Fig. 4:**
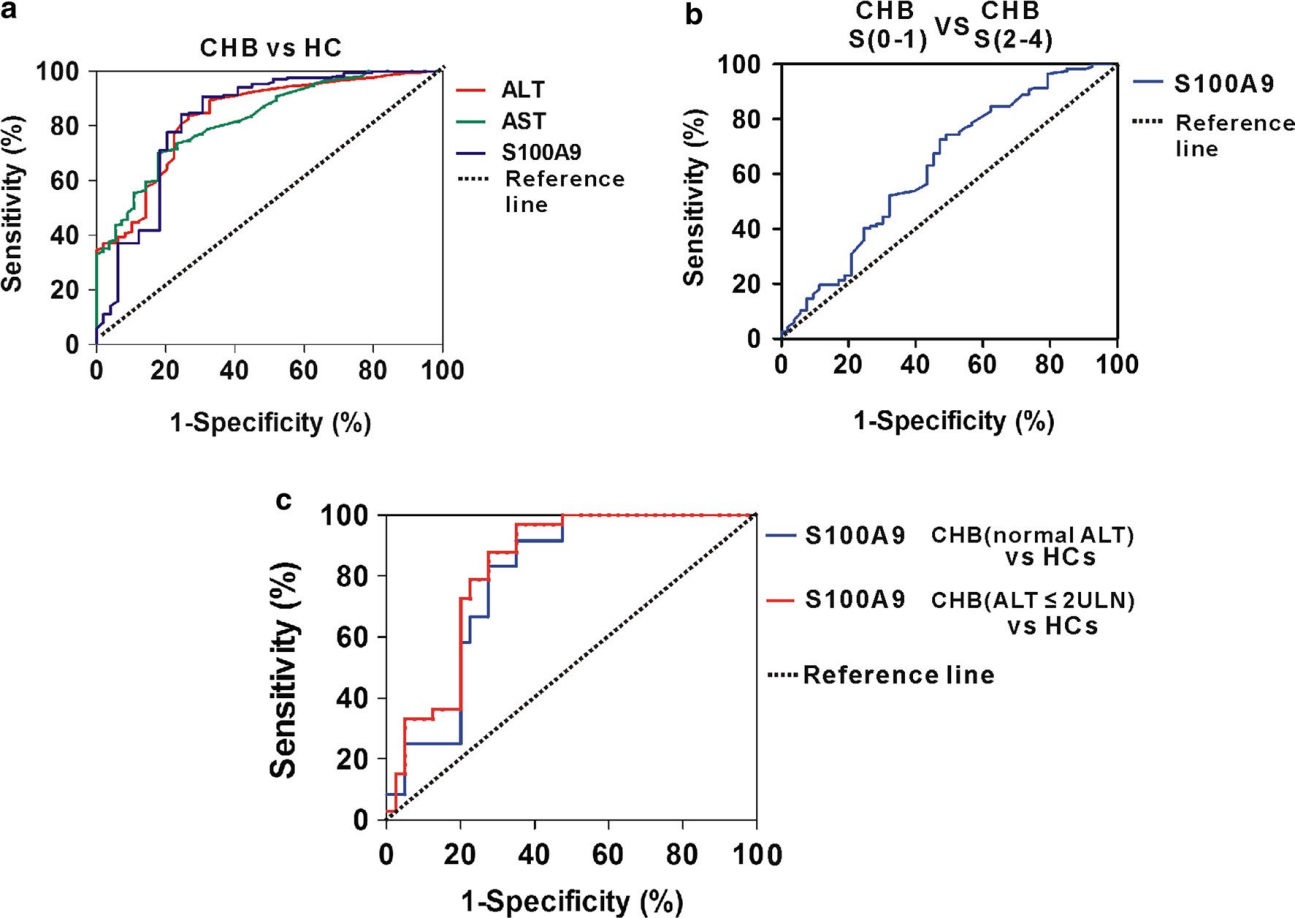
Differentiating power of S100A9 for liver necroinflammation. **a** ROC curves of serum ALT, AST and S100A9 for detecting liver necroinflammation in CHB patients from HCs. **b** ROC curve of serum S100A9 for detecting moderate-to-severe liver fibrosis from no or mild liver fibrosis in CHB patients. **c** ROC curves of serum S100A9 for detecting moderate-to-severe necroinflammation in CHB patients with normal or mildly increased ALT from HCs

ALT and AST are important biomarkers of liver necroinflammation and indicator of prior CHB treatment. Our study showed that ALT or AST had the high diagnostic value for identifying the patients with liver necroinflammation from healthy individuals, with an AUC of 0.836 (95% CI, 0.771–0.900) or 0.823 (95% CI, 0.765–0.883) (Fig. 4a). We next evaluated whether serum S100A9 can predict the patients with liver necroinflammation from healthy individuals. The ROC analysis indicated that diagnostic value of serum S100A9 was equivalent to ALT or AST, which yielded an AUC of 0.827 (95% CI, 0.749–0.904) with 71.2% sensitivity, 81.6% specificity and 76.4% accuracy (Fig. 4a). These findings indicate that the identified S100A9 could efficiently discriminate CHB patients from HCs. To further evaluate whether serum S100A9 can predict liver fibrosis grade, we compared serum S100A9 levels between CHB patients with moderate-to-severe fibrosis (S2–4) and with no or mild liver fibrosis (S0–1). ROC analysis showed that serum S100A9 levels had weaker diagnostic value for identifying liver moderate-to-severe fibrosis, which yielded an AUC of 0.631 (95% CI, 0.535–0.725) with 74.3% sensitivity, 50.9% specificity and 62.6% accuracy (Fig. 4b). These results imply weaker diagnostic performance of serum S100A9 for the detection of liver fibrosis than liver inflammation.

Although ALT is an important biomarker for liver necroinflammation, patients with normal or mildly increased ALT may have severe liver necroinflammation that may not be recognized without a liver biopsy. Therefore, we determined whether serum S100A9 levels could be used for differentiating moderate-to-severe necroinflammation (G ≥ 2). In patients with normal ALT levels, the area under the curve (AUC) of S100A9 for discriminating patients with moderate-to-severe necroinflammation (G ≥ 2) was 0.791 (95% CI, 0.670–0.913) with 91.7% sensitivity, 65.0% specificity and 78.3% accuracy (Fig. 4c). In patients with an ALT < 2 upper limit of normal (ULN), the AUC of S100A9 for discriminating patients with moderate-to-severe necroinflammation (G ≥ 2) was 0.826 (95% CI, 0.729–0.923) with 87.9% sensitivity, 72.5% specificity and 80.2% accuracy (Fig. 4c). These data suggest that serum S100A9 is superior to ALT for differentiating moderate-to-severe necroinflammation in patients with normal or minimally elevated ALT.

### Enhanced S100A9 expression in liver tissues and cells infected with HBV

**Fig. 5:**
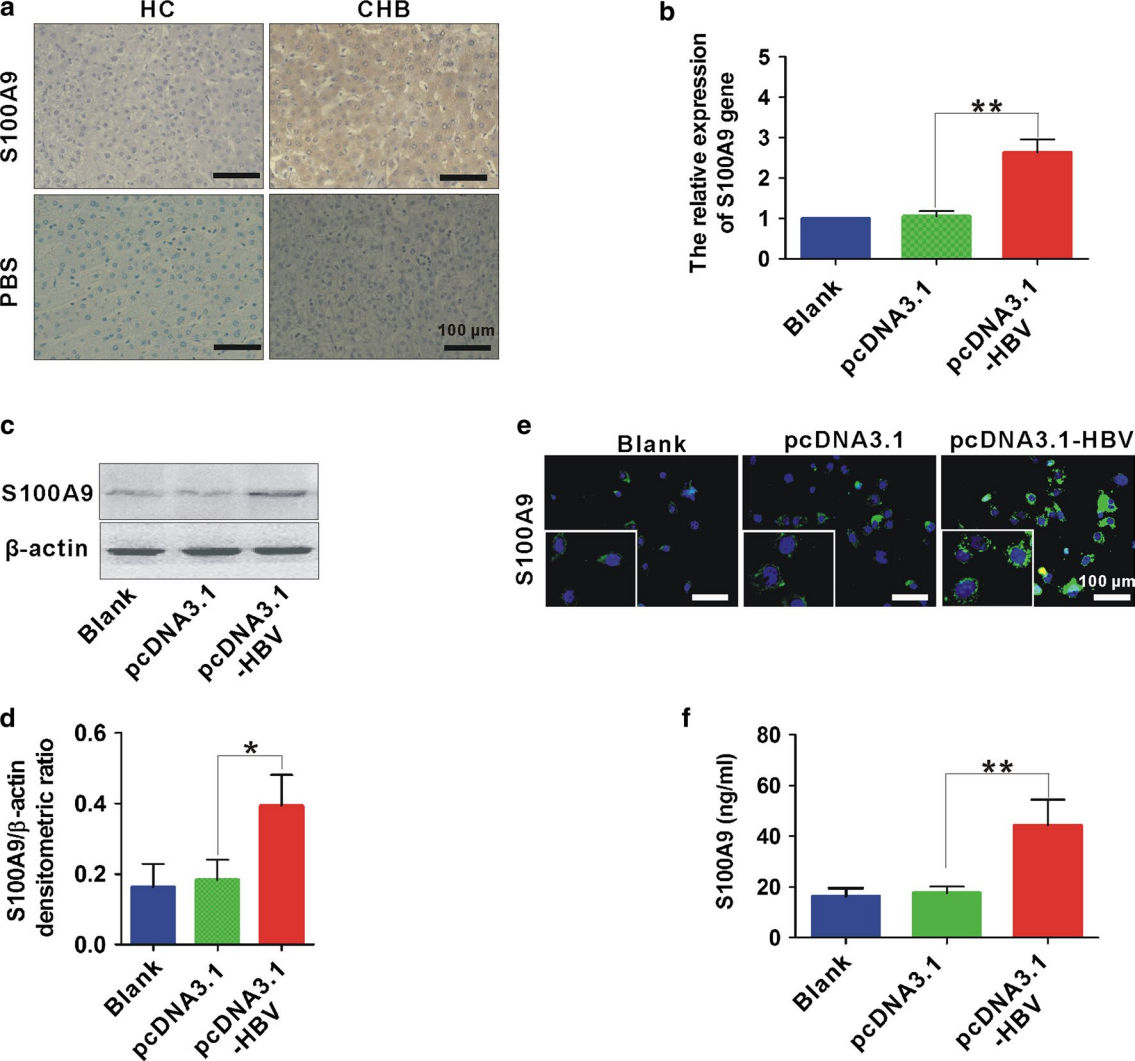
Elevated S100A9 expression in liver tissues and cells infected with HBV. **a** IHC staining of S100A9 in representative biopsied liver samples from CHB patients as well as the liver samples from HCs. Blank scale bars = 100 µm. **b** real-time PCR analysis of S100A9 gene in liver normal L02 cells transfected with and without pcDNA3.1-HBV or its control pcDNA3.1 for 24 h. **c** Western blot analysis of S100A9 expression in L02 cells transfected with and without pcDNA3.1-HBV or its control pcDNA3.1 for 48 h. **d** Statistical densitometric ratios of** c**. **e** Immunofluorescence staining for S100A9 in L02 cells transfected with and without pcDNA3.1-HBV or its control pcDNA3.1 for 48 h. White scale bars = 100 µm. **f** ELISA analysis for S100A9 levels in L02 cells transfected with and without pcDNA3.1-HBV or its control pcDNA3.1 for 48 h. Data represents the mean ± SD, *p < 0.05; **p < 0.01

Intrahepatic expression of S100A9 was detected by IHC in biopsied liver samples from HBV-infected patients as well as healthy controls that underwent liver biopsy for excluding malignancy. The enhanced immunoreactivity for S100A9 was detected in biopsied liver samples from randomly selected 12 patients with CHB patients (Fig. 5a). Further, the gene and protein expression of S100A9 was also up-regulated in HBV-transfected liver normal L02 cells compared to the cells transfected with control vector pcDNA3.1 by immunofluorescence staining and western blotting (Fig. 5b–d). And high levels of S100A9 was also observed in cell supernatant form HBV-transfected L02 cells by ELISA (Fig. 5e). These results suggest that HBV infection results in elevated S100A9 expression.

## Discussion

The cytoplasmic S100 proteins are promising new DAMPs in the pathogenesis of acute and chronic inflammation. S100A9 belongs to S100 protein family and exerts a proinflammatory role [23]. Several studies have been reported the correlation of S100A9 with inflammatory disease, including rheumatoid arthritis, inflammatory bowel disease and sepsis [24–26]. In the present study, we investigated the association of S100A9 with viral replication and liver necroinflammation in CHB patients, as well as the regulatory effect of HBV on S100A9 expression in vitro.

In our present study, serum S100A9 was correlated with viral load in CHB patients as well as in subgroups of HBeAg(−) immune reactivation phase and HBeAg(+) immune-active phase, which is consistent with previous reports showing that other DAMPs such as heat shock proteins have been reported to be supportive factors in the process of HBV replication [27–29]. Additionally, the IHC staining for S100A9 in tissues section from CHB patients also supported these clinical findings. We also demonstrated that HBV infection resulted in elevated S100A9 expression in liver cells. It is well known that the double-stranded DNA genome of HBV contains four overlapping open reading frames that encode the surface protein, the core protein, a polymerase and the X protein (HBx) [30]. HBx is a multifunctional regulator protein, which does not bind directly to DNA, but exerts transcriptional activation by its interaction with nuclear transcription factors such as NF-kappa B (NF-κB), AP-1 and CREB [31]. A recent study indicates that transcription factor NF-κB can bind to the promoter of S100A9 and regulate its expression in HCC [32]. So whether HBx-induced NF-κB activation is involved in the elevated S100A9 expression by HBV infection remained to be evaluated in the future study. One thing to note is that no correlation of serum S100A9 levels with viral loads was found in subgroup of in HBeAg(+) immune-tolerant phase. One possible reason is that small amounts of S100A9 protein were released into serum by hepatocytes due to the no or mild hepatic cell injury during this phase characterized by minimal necroinflammation and normal ALT.

Active hepatic necroinflammation is the dominative risk factor for developing live cirrhosis and HCC in CHB patients [33]. The latest guidelines also recommended that early control of liver necroinflammation should be the priority for the detection of liver fibrosis [34]. Thus, accurate evaluation of the initial stage of liver inflammation and progression represents a high priority and growing medical need. Recently, the majority of published serum biomarkers have been proposed for detecting significant liver fibrosis in CHB patients [35], only a few serum biomarkers were proposed to assist in the detection of liver necroinflammation in CHB patient [36, 37]. We then investigated whether serum S100A9 can be served as useful serum biomarker to evaluate the severity of hepatic necroinflammation in CHB patients. Positive correlation between S100A9 levels and the marker of liver necroinflammation in CHB patients found in the present study suggested an involvement of S100A9 in liver necroinflammation. The observation that the elevated S100A9 levels in patients with CHB gradually increased with severity of liver necroinflammation or fibrosis also supported this finding. Serum ALT has long been considered as the markers of liver necroinflammation and the indicators of antiviral therapy [4]. And ALT ≥ 2 ULN is used to indicate antiviral treatment according to the AASLD guideline for the CHB treatment [4]. However, ALT alone is inadequate to grade liver necroinflammation. Previous study in China showed that 49.2% of CHB patients with ALT < 2 ULN showed significant inflammation (G ≥ 2) and 36.4% showed significant fibrosis (S ≥ 2) [38]. Another biopsy report in US showed that 37% of CHB patients with normal ALT showed significant inflammation and significant fibrosis [39]. However, most patients refuse liver biopsy since it is an invasive and painful procedure, with rare but potential life-threatening complications. Therefore, there is a urgent need for several novel promising serum biomarkers to evaluate liver necroinflammation with higher diagnostic power. Although several noninvasive methods such as FibroScan have been recently used to assess the liver fibrosis status, it is not applicable to the evaluation for liver necroinflammation [40]. Our data showed that serum S100A9 levels are correlated with liver necroinflammation. Notably, it was also superior to ALT in differentiating grades of liver necroinflammation of CHB patients with normal or mildly elevated ALT. It could be meaningful that we could guess that if a CHB patient with higher serum S100A9 levels, the patient has a change to have significant necroinflammation even with normal or mildly elevated ALT. Even so, a larger number of patients with normal or mildly elevated ALT should be still included for verification in the future, confirming the diagnostic values in clinics.

It was known that persistent HBV infection is a major risk factor for cirrhosis and HCC. Long-term changes in serum levels of HBV DNA and ALT are independent predictors of risk for HCC. A strong correlation between serum ALT and intrahepatic HBV DNA levels has been reported in previous study, and both also correlates with the fibrosis staging [41]. Severe lobular necroinflammatory activity and more advanced fibrosis are also important predisposing factors for the development of HCC in CHB [42]. Our present study showed that S100A9 is correlated with both serum HBV DNA and ALT levels and also be regulated by HBV. In particular, our previous studies demonstrated that S100A9 was increased in HCC tumor tissues and cell lines compared to non-malignant controls and extracellular S100A9 protein could stimulate growth and invasion HCC cells by interaction with RAGE and activation of RAGE-dependent ERK1/2 and p38 MAPK signaling cascades [43, 44]. Given the correlation of S100A9 with HBV infection and previous finding of significance of S100A9 in HCC, S100A9 could be an important cancer promoter in HBV-related carcinogenesis and be a therapeutic target for patients with HBV-related HCC. In the future, the correlation between serum S100A9 and the risk of HCC would be investigated in patients with HBV-related chronic hepatitis.

Our study has several limitations. First, we did not evaluate the specificity of serum S100A9 for HBV infection. It would be better to compare HBV infection with other disease entities such as chronic hepatitis C or nonalcoholic steatohepatitis using the same set of analysis. Second, our study was carried out in Chinese patients who were characterized by Genotype B and C, further studies are required in CHB patients from different geographical areas or with different genotypes to confirm these data.

## Conclusions

In conclusion, the present studies demonstrate that HBV infection may enhance S100A9 expression in hepatocytes. Serum S100A9 levels from CHB patients are correlated with viral loads. Serum S100A9 may be a novel biomarker for liver necroinflammation, particularly in Chinese CHB patients with normal or mildly elevated ALT.

## Abbreviations

DAMP: damage associated molecular pattern
HBV: hepatitis B virus
HBx: HBV X protein
CHB: chronic hepatitis B
HCs: healthy controls
DAB: 3,3-diamino-benzidine tetrachloride
H2O2: hydrogen peroxide
NF-kappa B: NF-κB

## References

[1] Stasi C, Silvestri C, Voller F (2017). Emerging trends in epidemiology of hepatitis B virus infection. J Clin Transl Hepatol.

[2] Lee WM (1997). Hepatitis B virus infection. N Engl J Med.

[3] Levrero M, Zucman-Rossi J (2016). Mechanisms of HBV-induced hepatocellular carcinoma. J Hepatol.

[4] Lok AS, McMahon BJ (2007). Chronic hepatitis B. Hepatology.

[5] European Association For The Study Of The Liver (2012). EASL clinical practice guidelines: management of chronic hepatitis B virus infection. J Hepatol.

[6] Ravindran S, Hancox SH, Howlett DC (2016). Liver biopsy past, present and future. Br J Hosp Med.

[7] Regev A, Berho M, Jeffers LJ, Milikowski C, Molina EG, Pyrsopoulos NT, Feng ZZ, Reddy KR, Schiff ER (2002). Sampling error and intraobserver variation in liver biopsy in patients with chronic HCV infection. Am J Gastroenterol.

[8] Lidbury JA (2017). Getting the most out of liver biopsy. Vet Clin North Am Small Anim Pract..

[9] Pandolfi F, Altamura S, Frosali S, Conti P (2016). Key role of DAMP in inflammation, cancer, and tissue repair. Clin Ther.

[10] Venereau E, Ceriotti C, Bianchi ME (2015). DAMPs from cell death to new life. Front Immunol..

[11] Yang Q, Shi Y, Yang Y, Lou G, Chen Z (2015). The sterile inflammation in the exacerbation of HBV-associated liver injury. Mediators Inflamm.

[12] Wiersinga WJ, Leopold SJ, Cranendonk DR, van der Poll T (2014). Host innate immune responses to sepsis. Virulence..

[13] Kang R, Lotze MT, Zeh HJ, Billiar TR, Tang D (2014). Cell death and DAMPs in acute pancreatitis. Mol Med.

[14] Goh FG, Midwood KS (2012). Intrinsic danger: activation of toll-like receptors in rheumatoid arthritis. Rheumatology.

[15] Boyapati RK, Rossi AG, Satsangi J, Ho GT (2016). Gut mucosal DAMPs in IBD: from mechanisms to therapeutic implications. Mucosal Immunol.

[16] Fucikova J, Moserova I, Urbanova L, Bezu L, Kepp O, Cremer I, Salek C, Strnad P, Kroemer G, Galluzzi L, Spisek R (2015). Prognostic and predictive value of DAMPs and DAMP-associated processes in cancer. Front Immunol..

[17] Chen B, Miller AL, Rebelatto M, Brewah Y, Rowe DC, Clarke L, Czapiga M, Rosenthal K, Imamichi T, Chen Y (2015). S100A9 induced inflammatory responses are mediated by distinct damage associated molecular patterns (DAMP) receptors in vitro and in vivo. PLoS ONE.

[18] Srikrishna G (2012). S100A8 and S100A9 new insights into their roles in malignancy. J Innate Immun..

[19] Markowitz J, Carson WE (2013). Review of S100A9 biology and its role in cancer. Biochim Biophys Acta.

[20] Gebhardt C, Nemeth J, Angel P, Hess J (2006). S100A8 and S100A9 in inflammation and cancer. Biochem Pharmacol.

[21] Terrault NA, Bzowej NH, Chang KM, Hwang JP, Jonas MM, Murad MH, American Association for the Study of Liver D (2016). AASLD guidelines for treatment of chronic hepatitis B. Hepatology.

[22] Desmet VJ, Gerber M, Hoofnagle JH, Manns M, Scheuer PJ (1994). Classification of chronic hepatitis: diagnosis, grading and staging. Hepatology.

[23] Foell D, Wittkowski H, Vogl T, Roth J (2007). S100 proteins expressed in phagocytes: a novel group of damage-associated molecular pattern molecules. J Leukoc Biol.

[24] Baillet A (2010). S100A8, S100A9 and S100A12 proteins in rheumatoid arthritis. Rev Med Interne..

[25] Leach ST, Yang Z, Messina I, Song C, Geczy CL, Cunningham AM, Day AS (2007). Serum and mucosal S100 proteins, calprotectin (S100A8/S100A9) and S100A12, are elevated at diagnosis in children with inflammatory bowel disease. Scand J Gastroenterol.

[26] Gao S, Yang Y, Fu Y, Guo W, Liu G (2015). Diagnostic and prognostic value of myeloid-related protein complex 8/14 for sepsis. Am J Emerg Med.

[27] Wang YP, Liu F, He HW, Han YX, Peng ZG, Li BW, You XF, Song DQ, Li ZR, Yu LY (2010). Heat stress cognate 70 host protein as a potential drug target against drug resistance in hepatitis B virus. Antimicrob Agents Chemother.

[28] Hu J, Flores D, Toft D, Wang X, Nguyen D (2004). Requirement of heat shock protein 90 for human hepatitis B virus reverse transcriptase function. J Virol.

[29] Zhou YB, Wang YF, Zhang Y, Zheng LY, Yang XA, Wang N, Jiang JH, Ma F, Yin DT, Sun CY, Wang QD (2012). In vitro activity of cepharanthine hydrochloride against clinical wild-type and lamivudine-resistant hepatitis B virus isolates. Eur J Pharmacol.

[30] Arzumanyan A, Reis HM, Feitelson MA (2013). Pathogenic mechanisms in HBV- and HCV-associated hepatocellular carcinoma. Nat Rev Cancer.

[31] Diao J, Garces R, Richardson CD (2001). X protein of hepatitis B virus modulates cytokine and growth factor related signal transduction pathways during the course of viral infections and hepatocarcinogenesis. Cytokine Growth Factor Rev.

[32] Nemeth J, Stein I, Haag D, Riehl A, Longerich T, Horwitz E, Breuhahn K, Gebhardt C, Schirmacher P, Hahn M (2009). S100A8 and S100A9 are novel nuclear factor kappa B target genes during malignant progression of murine and human liver carcinogenesis. Hepatology.

[33] Sarin SK, Kumar M, Lau GK, Abbas Z, Chan HL, Chen CJ, Chen DS, Chen HL, Chen PJ, Chien RN (2016). Asian-Pacific clinical practice guidelines on the management of hepatitis B: a 2015 update. Hepatol Int.

[34] European Association for the Study of the Liver (2017). EASL 2017 Clinical Practice Guidelines on the management of hepatitis B virus infection. J Hepatol.

[35] Castera L (2012). Noninvasive methods to assess liver disease in patients with hepatitis B or C. Gastroenterology.

[36] Mohamadnejad M, Montazeri G, Fazlollahi A, Zamani F, Nasiri J, Nobakht H, Forouzanfar MH, Abedian S, Tavangar SM, Mohamadkhani A (2006). Noninvasive markers of liver fibrosis and inflammation in chronic hepatitis B-virus related liver disease. Am J Gastroenterol.

[37] Wang JY, Mao RC, Zhang YM, Zhang YJ, Liu HY, Qin YL, Lu MJ, Zhang JM (2015). Serum microRNA-124 is a novel biomarker for liver necroinflammation in patients with chronic hepatitis B virus infection. J Viral Hepat.

[38] Chen EQ, Huang FJ, He LL, Bai L, Wang LC, Zhou TY, Lei XZ, Liu C, Tang H (2010). Histological changes in Chinese chronic hepatitis B patients with ALT lower than two times upper limits of normal. Dig Dis Sci.

[39] Lai M, Hyatt BJ, Nasser I, Curry M, Afdhal NH (2007). The clinical significance of persistently normal ALT in chronic hepatitis B infection. J Hepatol.

[40] Rizzo L, Calvaruso V, Cacopardo B, Alessi N, Attanasio M, Petta S, Fatuzzo F, Montineri A, Mazzola A, L’Abbate L (2011). Comparison of transient elastography and acoustic radiation force impulse for non-invasive staging of liver fibrosis in patients with chronic hepatitis C. Am J Gastroenterol.

[41] Wong DK, Yuen MF, Tse E, Yuan H, Sum SS, Hui CK, Lai CL (2004). Detection of intrahepatic hepatitis B virus DNA and correlation with hepatic necroinflammation and fibrosis. J Clin Microbiol.

[42] Lee SH, Chung YH, Kim JA, Jin YJ, Park WH, Choi JG, Lee D, Shim JH, Yu E, Jang MK (2011). Histological characteristics predisposing to development of hepatocellular carcinoma in patients with chronic hepatitis B. J Clin Pathol.

[43] Wu R, Duan L, Ye L, Wang H, Yang X, Zhang Y, Chen X, Zhang Y, Weng Y, Luo J (2013). S100A9 promotes the proliferation and invasion of HepG2 hepatocellular carcinoma cells via the activation of the MAPK signaling pathway. Int J Oncol.

[44] Wu R, Duan L, Cui F, Cao J, Xiang Y, Tang Y, Zhou L (2015). S100A9 promotes human hepatocellular carcinoma cell growth and invasion through RAGE-mediated ERK1/2 and p38 MAPK pathways. Exp Cell Res.

